# Left-sided appendicitis in intestinal malrotation: a minimally invasive approach

**DOI:** 10.1093/jscr/rjac274

**Published:** 2022-06-16

**Authors:** Gerard Feeney, Enda Hannan, Mohammed Alagha, Yasser Abdeldaim

**Affiliations:** Department of Surgery, University Hospital Limerick, Limerick, Ireland; Department of Surgery, University Hospital Limerick, Limerick, Ireland; Department of Surgery, University Hospital Limerick, Limerick, Ireland; Department of Surgery, University Hospital Limerick, Limerick, Ireland

**Keywords:** acute appendicitis, intestinal malrotation, laparoscopic appendicectomy, left-sided appendicitis, midgut malrotation

## Abstract

Intestinal malrotation is a rare clinical entity that occurs in 1/6000 live births. Acute appendicitis (AA) is commonly recognized clinically by migratory right iliac fossa pain. We present a rare case of AA in a patient with previously undiagnosed IM that posed a diagnostic challenge due to abnormal caecal location, which was managed by a laparoscopic approach. The presence of undiagnosed congenital anomalies such as IM can render diagnosis of even seemingly straightforward conditions such as AA challenging, meaning that the presence of classical clinical findings cannot always be relied upon. One should have a low threshold for performing cross-sectional imaging in cases where clinical findings do not yield a satisfactory diagnosis. The adult patient with AA in the context of incidental type 1 IM can be managed laparoscopically by a simple modification of standard technique, without the need to correct malrotation, thus allowing the patient to benefit minimally invasive surgery.

## INTRODUCTION

Acute appendicitis (AA) is frequently associated with a classical presentation of abdominal pain that originates centrally before localizing to the right iliac fossa [1]. When a patient presents with this textbook pattern, the diagnosis of AA is straightforward, with many surgeons relying on clinical features alone to justify laparoscopy without preoperative cross-sectional imaging [1]. However, an absence of this typical picture may result in a missed diagnosis, which carries significant risk of morbidity and mortality. AA is the most common condition requiring emergency abdominal surgery [1]. Thus, a high index of suspicion should be maintained for any patient with acute abdominal pain.

Intestinal malrotation (IM) is a congenital anomaly referring to the nonrotation or incomplete rotation of the intestines around the axis of the superior mesenteric artery (SMA) during foetal development [2]. There are three different types of IM: type 1 (nonrotation), type 2 (duodenal malrotation) and type 3 (duodenal and caecal malrotation) [2]. IM is rare with an incidence of one in 6000 live births [2]. The majority will present acutely within the first month of life with obstructive symptoms [2]. However, a small minority are asymptomatic and thus undiagnosed [2]. Such patients are equally at risk of AA as the normal population; however, the atypical location of the caecum may render the diagnosis challenging. We present the case of AA in a patient with previously undiagnosed IM to highlight the diagnostic and therapeutic challenges encountered in this rare clinical entity.

## CASE REPORT

A 47-year-old female with no significant past medical history presented to the emergency department with a one-day history of left iliac fossa (LIF) pain. Her abdomen was tender and guarded in the LIF. Baseline laboratory investigations revealed raised inflammatory markers. An ultrasound of the abdomen was performed, which revealed no demonstrable cause for the clinical picture. This was followed by computed tomography (CT) of the abdomen and pelvis. This revealed the presence of a type 1 IM, with the small bowel on the right side of the abdomen, the large bowel on the left and an inversion of the normal anatomical relationship of the SMA and superior mesenteric vein (SMV). A dilated blind-ending tubular structure that was 1.6 cm in diameter with associated fat-stranding and free fluid was noted in the mid-abdomen and was arising from the left-sided caecum, in keeping with a diagnosis of AA.

**Figure 1:**
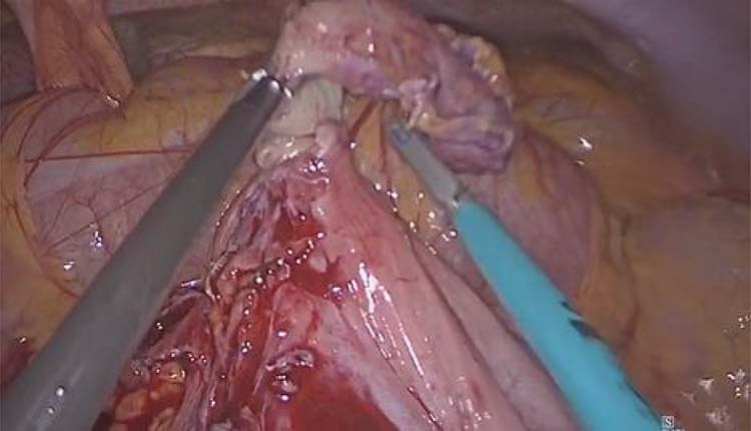
Left-sided caecum as a result of IM with AA.

The patient underwent surgical exploration by laparoscopy via a 12-mm balloon port placed subumbilical by Hasson technique. Given the abnormal location of the caecum, the standard laparoscopic port placement for an appendicectomy was modified accordingly, with two 5-mm ports inserted under laparoscopic vision in the right iliac fossa (RIF) and suprapubic region, respectively, allowing for optimal triangulation of the target anatomy. A thorough diagnostic laparoscopy concurred with the radiological findings, with a small bowel walk from the right-sided duodenojejunal flexure to the left-sided terminal ileum yielding no further abnormality (Fig. 1). Following division of the mesoappendix, the appendix was divided at the base following placement of three Endoloops^®^ (two proximal, one distal) and extracted via the subumbilical incision by a specimen retrieval bag. The patient made an uneventful recovery and remains well at postoperative follow-up, with histopathological analysis confirming AA.

## DISCUSSION

In this case, a common condition that would usually pose no diagnostic or therapeutic challenge to the experienced surgeon overlapped with an exceedingly rare developmental anomaly, creating a clinical scenario that posed high risk of a potentially devastating delay in diagnosis. It is essential to be aware that, even in the context of normal anatomy, the classical clinical picture of migratory RIF pain and associated abdominal signs only occurs in 50% of patients [1]. Accordingly, the rate of missed diagnosis of appendicitis has been reported to be as high as 23.5% of cases in adult emergency departments, which is alarmingly high for the most common abdominal emergency [1]. While clinical examination, scoring systems such as the Alvarado score and the use of focused ultrasonography are often useful in aiding diagnosis, all of these measures could have falsely reassured the clinician in the described case due to the abnormal location of the caecum [1]. In this case, CT was essential in ensuring a timely diagnosis in a case where an unpredictable anatomical anomaly obscured the clinical picture, which highlights the importance of cross-sectional imaging in a case where history and examination does not yield a satisfactory diagnosis.

While AA in IM has been described in a small number of previously published cases, many of these were managed by laparotomy [3–5]. The indication for laparotomy varied. In a number of cases, a laparotomy was performed with the intention of correcting the malrotation as well as performing an appendectomy [3–5]. However, in the adult patient with an asymptomatic type 1 IM, surgical repair is not indicated [2, 3]. In fact, the Ladd procedure for correction of IM typically concludes with placement of small bowel on the right abdomen and the colon on the left, which is the configuration encountered in type 1 IM [2]. In other reported cases, the indication for laparotomy included clinical status in delayed diagnosis, the atypical caecal location and lack of expertise in laparoscopic surgery [3–5]. Our case is unique by highlighting an easily reproducible approach to laparoscopic appendectomy in AA, and demonstrates that surgical correction of the congenital anomaly is not indicated in the asymptomatic adult with type 1 IM. This allows the patient to benefit from minimally invasive surgery, with a shorter recovery time, less postoperative pain and improved cosmesis.

In conclusion, the presence of undiagnosed congenital anatomical variations such as IM can make the diagnosis of even the most seemingly straightforward conditions such as AA challenging, meaning that the presence of classical clinical findings cannot always be relied upon. Thus, one should have a low threshold for performing cross-sectional imaging in cases where history and examination does not yield a satisfactory diagnosis. The adult patient with AA in the context of incidental type 1 IM can be managed laparoscopically by a simple modification of standard technique, without the need to correct malrotation, thus allowing the patient to benefit from the advantages of minimally invasive surgery.

## CONFLICT OF INTEREST STATEMENT

None to declare.

## FUNDING

No funding received.

## CONSENT

Obtained from the patient and documented in the medical notes.
